# Timing of Gun Fire Influences Sprinters’ Multiple Joint Reaction Times of Whole Body in Block Start

**DOI:** 10.3389/fpsyg.2017.00810

**Published:** 2017-05-18

**Authors:** Mitsuo Otsuka, Toshiyuki Kurihara, Tadao Isaka

**Affiliations:** Faculty of Sport and Health Science, Ritsumeikan UniversityKusatsu, Japan

**Keywords:** warning signal, imperative signal, foreperiod, GRF, motion capture

## Abstract

Experienced sprinters are specifically adapted to pre-planning an advanced motor program. Herein, sprinters are able to immediately accelerate their center of mass forward with a whole-body coordinated motion, following a steady state crouched position. We examined the effect of variable timing of reaction signals on multiple joint reaction times (RT) and whole-body RT for specialist sprinters. Twenty well-experienced male sprinters performed five start-dashes from a block start under five variable foreperiod (FP) length conditions (1.465, 1.622, 1.780, 1.938, and 2.096 s), with trials randomly timed between a warning and an imperative tone. Participants’ sprinting motion and ground reaction forces of their four limbs during the block start were measured simultaneously. Whole-body RT was significantly shorter when FP length was longer; the values of whole-body RT were 117 ± 5 ms, 129 ± 5 ms, 125 ± 4 ms, 133 ± 6 ms, and 156 ± 8 ms in the 2.096, 1.938, 1.780, 1.622, and 1.465-s FP-length conditions, respectively. A repeated-measures analysis of variance found a significant joint-by-FP length interaction in joint-moment RT. These findings suggest that FP length affects coordinated motion in four limbs and whole-body RT. This information will be able to lead to new methods for start signals in sprint running events and advance our understanding of the association between FP length and dynamic coordinated motion.

## Introduction

Experienced sprinters are specifically adapted to accelerating their center of mass immediately forward after being in a steady set crouched position (Otsuka et al., 2014). This block start motion sequence consists of eight steps: first, crouching down for the ‘on your marks’ position with legs flexed markedly on two starting blocks and with hands on the ground, second, being steady in the ‘on your marks’ position, third, hearing a warning signal, the ‘set’ command, fourth, extending legs for the crouched ‘set’ position, fifth, being in the ‘set’ position in which the forward arms crutch position is important for subsequent acceleration (Slawinski et al., 2010), sixth, hearing an imperative signal, i.e., gunfire, seventh, taking off the hands and extending legs while taking off the rear foot and the front foot from block starts, respectively, and eighth, connecting to the subsequent acceleration phase in which sprinters run with a forward-leaned posture (e.g., Kugler and Janshen, 2010; Morin et al., 2011, 2012). During the fourth- and fifth-step motions, an advanced motor program such as a whole-body acceleration motion in the seventh and eighth steps would be pre-planned. This type of prepared and advanced motor program is physiologically evoked in the neuromuscular system after a loud sound such as a start gun (Maslovat et al., 2008).

Reaction time (RT), which is the duration from response signal onset to the first discernible change in focal force-generation activity, is influenced by the anticipation of the timing of the stimulus input (e.g., Niemi, 1979; Niemi and Näätänen, 1981). The RT is relatively longer after a shorter foreperiod (FP), which is the duration between warning and imperative signals and ranges from 0.5 to 3.0 s. This is due to the fact that involving a delay in RT to an imperative signal by stimuli that are too closely spaced (psychological refractory period, e.g., Telford, 1931) and by unplanning the subsequent motor program.

RT in block start has already been researched in the past (e.g., Henry, 1952; Gutiérrez-Dávila et al., 2006; Lipps et al., 2011). In a competitive race, experienced sprinters immediately react to auditory stimuli, a start gun, with a whole-body motion. The starter subjectively determines the gun-signal timing after checking that all sprinters are steady in a crouched set position; therefore, the duration from ‘set’ to the gun signal is different across races. The set and gun signals of the starter can be regarded as the warning signal and imperative signal, respectively, in a simple auditory reaction task (Brown et al., 2008). A few previous studies on block start reported the length of FP: 2.0 s of Brown et al. (2008) and 3.0-4.0 s of Pain and Hibbs (2007). However, these FP lengths were not strictly determined, and did not refer to the distribution of FP in an athletic race. It has been reported that the RT was 166 ± 30 ms (mean ± SD) for male sprinters in a competitive race (Tønnessen et al., 2013); however, the RT was not classified by various FP lengths. To our knowledge, few previous basic studies reported the RT under various FP lengths whose imperative signal was a loud auditory stimulus, which can be considered a startle stimulus psychologically. Therefore, it is difficult to show a standard RT based on FP lengths through various previous studies. Only Cressman et al. (2006) clarified that when FP lengths range from 2.5 to 5.5 s with a startle stimulus, the electromyographic (EMG) latency of a wrist extensor muscle does not change (Cressman et al., 2006). This suggests that an intended motion is programmed prior to the arrival of the imperative go stimulus within 2.5 s of the warning stimulus and is released with a startle response regardless of the FP length. In block start, an additional motion, extending legs for the crouched ‘set’ position, is involved during the first part of the FP. Moreover, because the FP length was set at 2.0 s in one previous study (Brown et al., 2008), in a competitive race, there is a possibility that a starter’s gunfire timing of a gunfire is shorter than that in the study mentioned previously (Cressman et al., 2006). Therefore, there is a possibility that short FP lengths observed in a competitive race do not allow sprinters to prepare an acceleration motor program and delay the subsequent RT.

Previous basic studies measuring surface EMG activity have revealed that the optimal FP length (with the shortest EMG latency) differs in accordance with the muscle (Yuanhui and Kasai, 1993; Cuisinier et al., 2007). For instance, in a single upper-limb movement, FP length affects EMG latencies for both the elector spinaes and contralateral tensor fascia lata; however, FP length does not affect latencies of both biceps femoris. This may indicate that a coordination pattern of the muscle changes based on the FP length. In basic studies, a simple reaction task under variable FP length was conducted with a single/partial joint task (e.g., Niemi, 1979; Niemi and Näätänen, 1981). In contrast, in block start, the RT is generated by a whole-body coordinated motion. Whole-body RT emerges from coordinated movement by force generation in multiple joints of all limbs. Therefore, it is necessary to clarify the effect of FP length on whole-body RT within a dynamic initial motion (e.g., a block start), which would be affected by different multiple-joint coordination patterns for all limbs based on FP length.

The purpose of this study was to clarify the effect of variable FP length in the block start signal on subsequent whole-body coordinated pattern and whole-body RT for experienced sprinters. This information may provide evidence-based advice for sprinters, coaches, and organizations building a common athletic regulation. Our hypothesis was that longer FP length in the block start signal based on recent athletic races would affect whole-body coordinated pattern and shorter whole-body RT.

## Materials and Methods

### Participants

Twenty male sprinters participated in this sprint start experiment (mean ± standard error [SE], age: 21.9 ± 2.1 years; height: 177.1 ± 1.2 cm; body mass: 67.9 ± 1.2 kg; 100-m personal record: 10.85 ± 0.10 s; training duration: 9.6 ± 0.5 years). Seven participants participated in the World Championships (*n* = 2), Summer Universiade (*n* = 2), and similar-level international games (*n* = 3). All experiments were conducted after obtaining approval from the Research Ethics Committee involving Living Human Participants in Biwako Kusatsu campus, Ritsumeikan University; this study was performed in accordance with the ethical guidelines of the Declaration of Helsinki. All participants gave written informed consent before participating in the study.

### Auditory Stimuli

**FIGURE 1:**
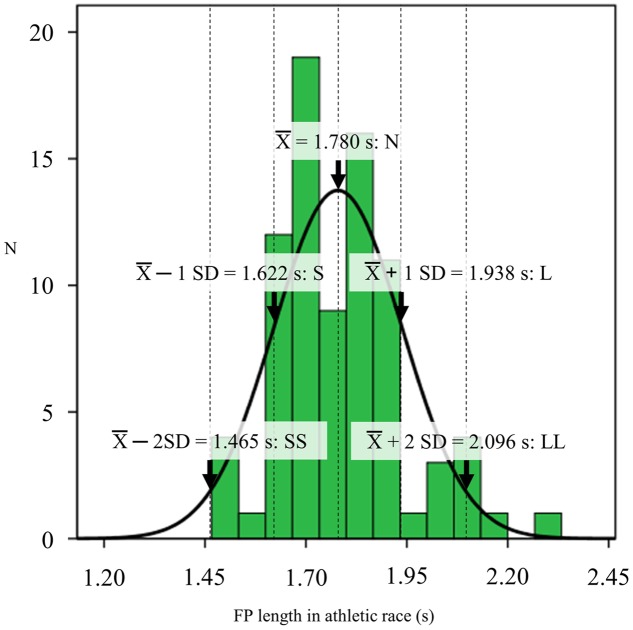
**Histogram of foreperiod (FP) length in recent international races over the last 5 years**. The black line shows its normal distribution curve. The five FP lengths for further experiments were determined using the mean, mean ± 1 SD and mean ± 2 SD (1.465, 1.622, 1.780, 1.938, and 2.096 s).

Foreperiod lengths were determined based on a distribution of FP in recent athletic races. To examine the distribution of variable FP lengths in athletic games, a total of 88 male and female large-scale 100-m races over the last 5 years were recorded from publicly available television broadcasts. The competitions included the 2012 Olympics and the World Championships in 2011, 2013, and 2015. The auditory data were collected from heats to finals. The races whose set and start signal could be clearly heard without the voice of the announcer were analyzed, for a total of 83 races. These FP lengths were analyzed by intervals of 10 ms using sound editing software (EDIUS Neo 3 version 3.05, Grass Valley Inc., Hyogo, Japan). Onsets of the starter’s set and gun signals were visually determined by sound wave information. **Figure 1** shows the distribution of FP length in recent international races. The FP length of the start signal was 1.780 ± 0.017 s (*SD*: 0.158 s). Based on the distribution of the measured FP length, five FP lengths for the experiment were determined by the mean and SD: mean – 2 SD (1.465 s), mean – 1 SD (1.622 s), mean (1.780 s), mean + 1 SD (1.938 s) and mean + 2 SD (2.096 s) (SS, S, N, L, and LL conditions, respectively). The warning and imperative tones were generated using Matlab (R2011a version 7.12.0.635, The MathWorks, Inc., Natick, MA, US) and a trumpet horn (XB-11S, Hangzhou Xingbo Electric Sound Equipment Co., Hangzhou, China) placed beside the participants approximately 30 cm from the ear. The warning and imperative tones were set with intensities of 70 and 120 dB, respectively, which were used in a previous study on startle response in a block start (Brown et al., 2008). These intensities were calibrated using a sound level meter (RAMA11008, Thanko Inc., Tokyo, Japan).

### Experimental Trial

The experimental trial involved five 5-m dashes from a block start under a variable FP-length condition with the trial randomly timed between the warning and imperative tones. Participants were instructed to start as quickly and strongly as possible without anticipating the go signal. The rest duration between trials was 3 min. In order to avoid participants from accurately anticipating the go signal timing, a catch trial in which the go signal was not delivered was used (Brown et al., 2008). The catch trial incidence was 20%. Before the experiment, participants were familiarized using three submaximal repetitions at SS-, N- and LL-FP trials (similar rest durations), following self-warming up for >30 min. Participants were instructed to use their spike shoes and their preferred anteroposterior starting position.

### Data Measurement

**FIGURE 2:**
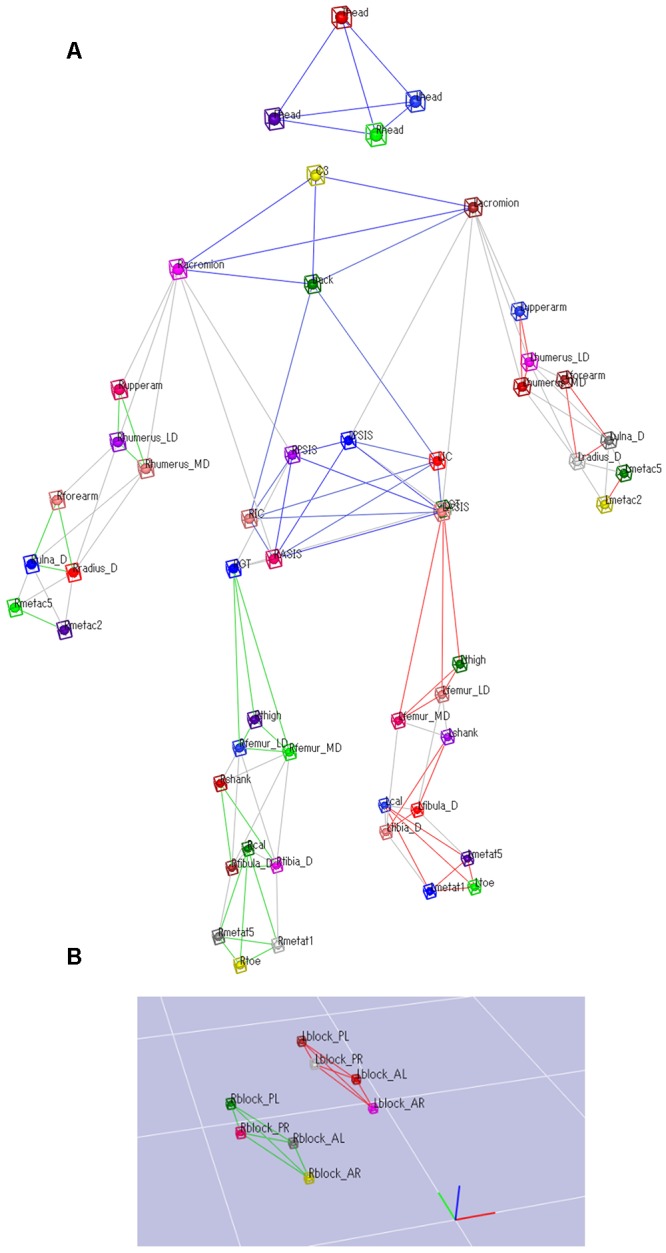
**Marker set attached to the whole body. (A)** A 52 retro-reflective markers were attached to the whole body. **(B)** A total of 8 retro-reflective markers were attached to the starting blocks.

**FIGURE 3:**
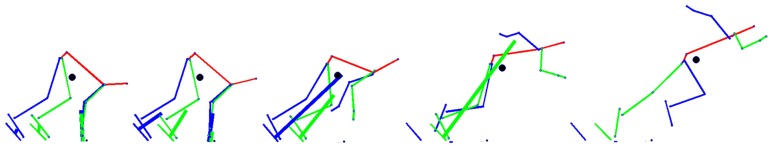
**A typical example of postures during the block start**. The green line shows the front-leg side (the arm was defined as the front-arm), the blue line shows the rear-leg side (the arm was defined as the rear-arm), and the red line shows the head, trunk, and pelvis in the sagittal plane. The thin line shows the body segment vector of the sprinter and the thick line shows the ground reaction force (GRF) vector.

Data on the participants’ sprinting motion and ground reaction forces (GRF) during the block start were captured simultaneously. A total of 60 retro-reflective markers sized 12 mm were attached to the whole body and the starting blocks. **Figure 2** shows the detailed marker set for the whole body and starting blocks. The three-dimensional locations of the markers were recorded using a 16-camera motion capture system (Raptor-E digital; Motion Analysis Corporation, Santa Rosa, CA, United States) sampling at 250 Hz. The starting blocks were adjusted onto separate force plates (Mero et al., 2006; Otsuka et al., 2015). GRFs generated by the four upper and lower limbs, that is, front-leg, rear-leg, front-leg-side arm (we define the arm that moved forward during kicking starting blocks as the front-arm), and rear-leg-side arm (we define the arm that moved backward during kicking starting blocks as the rear-arm), were separately measured using a 3.0-m long force plate arranged in two adjacent rows (total of 10 force plates, 0.60 m × 0.40 m; TF-4060-B; Tech-Gihan Inc., Kyoto, Japan), sampling at 1000 Hz. **Figure 3** shows stick pictures describing the starting motion and GRF vectors during the block start.

### Data Analysis

A 15-segment rigid body model was created, with the head, trunk, pelvis, upper arms, forearms, hands, thighs, shanks, and feet. Segmental anthropometric data was used from Ae et al.’s (1992) value. The locations of the center of rotation of the hip were estimated from anatomical landmarks using a predictive approach (Bell et al., 1990), and that of the knee, ankle, elbow, and wrist used the midpoint between two reflective-marker positions attached around the joint. The location of the shoulder joint used the position of a reflective marker attached on the lateral position of the acromion. In a previous study, joint moments were calculated using GRF data captured on a force plate under the block (Mero et al., 2006). Accordingly, the center of pressure on the starting blocks in the sagittal plane was determined by projecting the intersection of a GRF vector and inclination vector of the starting block in the sagittal plane. The marker trajectories and GRF data were filtered using a fourth-order zero-lag low-pass Butterworth filter with a cut-off frequency of 10 and 25 Hz, respectively. Joint moments of the shoulder, elbow, wrist, hip, knee, and ankle in the sagittal plane were calculated using a standard inverse dynamics approach (Winter, 2009).

**FIGURE 4:**
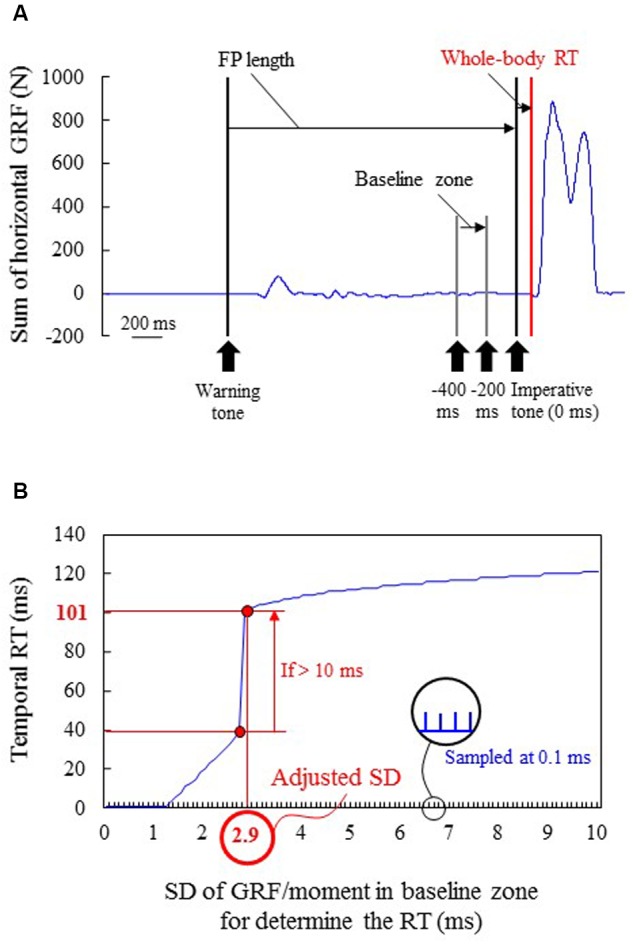
**A typical example of determining the actual reaction time (RT) in block starts. (A)** Time-series sum of horizontal GRF of the upper and lower limbs. The vertical black line shows the timing of the warning and imperative tones. Five FP lengths were prepared in accordance with the distribution of FP in athletic races. **(B)** Each standard deviation (SD) of GRF or moment in baseline zone (-400 to -200 ms from the imperative tone) for determining the onset threshold for the temporal RT using the SD. This SD was increased sampling at 0.1 ms. The actual RT was determined if the difference between the temporal RT and the next temporal RT was over 10 ms. In this typical example, mean value + 2.9 SD (adjusted SD) was the most appropriate threshold because the smaller SD (<2.9) failed to determine the actual 101 ms RT; the temporal RT were less than 40 ms. In this method, error can be found by checking whether differences are over 10 ms between the temporal RT and the next temporal RT added 0.1 SD.

The RT generated by the sum of the GRFs of the upper and lower limbs (whole-body RT) and the RT generated by the moment in each joint (joint RT) were calculated using a threshold of the mean value ± the adjusted SD, which differed based on each participant and each joint, in the baseline zone (from -400 to -200 ms of the imperative tone; **Figure 4A**). Because the variability of time-series data of sum of GRFs or joint moment in the baseline zone was different based on individual differences and joints, a fixed SD was inappropriate to determine the onset instant of the pushing blocks or floor. Therefore, the SDs were adjusted in accordance with each trial; temporal RT was first determined by increasing the SD sampled at 0.1 ms, and the actual RT was determined if the difference between the temporal RT and the next temporal RT was over 10 ms (**Figure 4B**). When GRF/joint moment data increased from the baseline (following a sufficient decrease), the GRF/moment threshold value was determined during the decrease. If the temporal RT was not clearly appropriate for the actual RT (e.g., <60 ms) using the detection method, the actual RT was finally checked by visually locating and manually adjusting the onset mark to the point at which the activity first increased/decreased.

### Statistical Analysis

Data are presented as mean ± standard error (SE). All datasets were initially checked for normality and homogeneity of variance. To assess changes in whole-body RT and changes in joint RT within the same joint across the five FP-length conditions, a repeated-measures analysis of variance (ANOVA) was conducted. With regard to joint RT, a two-way repeated-measures ANOVA was conducted with FP length and joint as factors. The FP length × joint interaction was to clarify whether changes in the joint RT in the five conditions were different based on the joints and to assess the multi-joint coordination pattern in each condition. Bonferroni’s test was used in all *post hoc* comparisons in all ANOVA. In each condition, Pearson’s correlation coefficients were used to assess the relationships between whole-body RT and joint RT. In each condition, stepwise multiple regression analyses were conducted to identify the joint RT that were closely related to whole-body RT and to assess the relationship between the whole-body coordinated pattern and whole-body RT. Statistical significance was determined at *P* < 0.05 and the *P*-value was adjusted for the number of comparisons in *post hoc* comparisons in all ANOVA. Using partial η^2^, effect sizes were calculated for each main effect for all repeated-measures ANOVA. Data were normally distributed and statistical analyses were performed using SPSS version 19.0.0 (SPSS Inc., Chicago, IL, United States).

## Results

**FIGURE 5:**
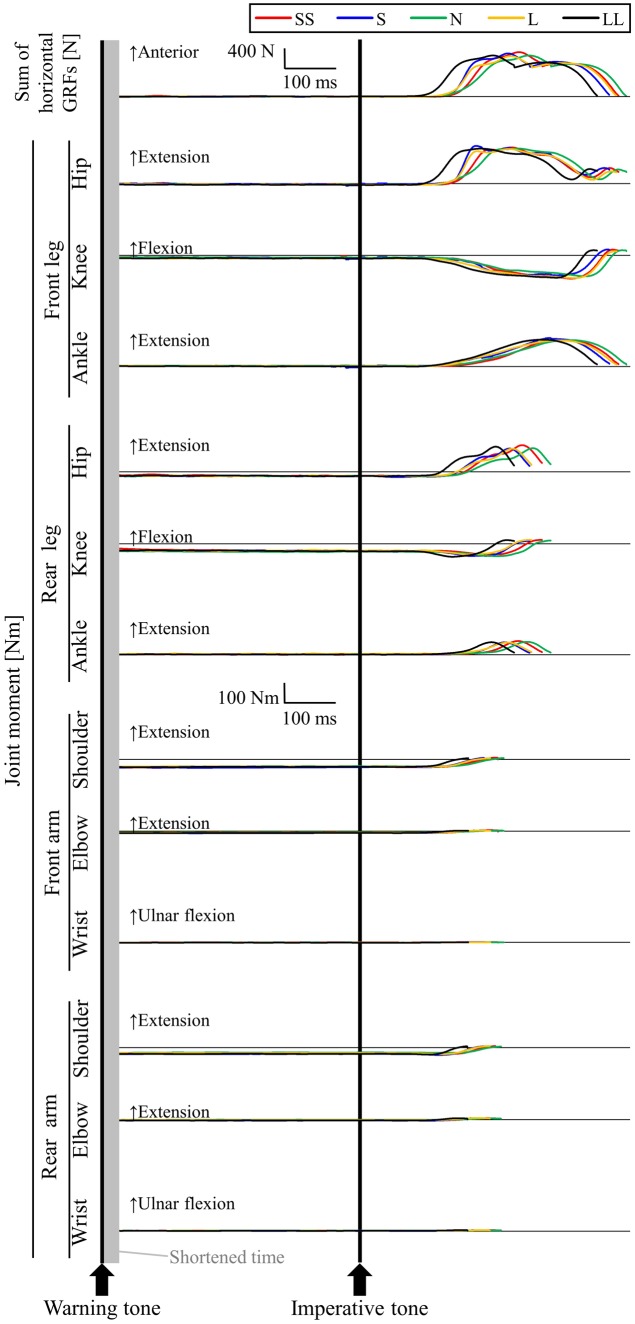
**Typical example of the sum of horizontal GRF and joint moments for participant #11 in the five FP-length conditions**. SS, S, N, L, and LL were conditions whose FP lengths were 1.465, 1.622, 1.780, 1.938, and 2.096 s, respectively.

**Figure 5** shows a typical example of whole-body RT and RT of joint moments during the stance phase in the five conditions (participant #11). For the LL condition, after the gun signal, the front-arm shoulder, already generating a flexion moment at the set position, was the first to react (joint RT: 61 ms), reducing the flexion moment. A similar time-moment curve was observed in the rear-arm shoulder; however, joint RT was delayed compared to the front-arm shoulder (joint RT: 123 ms). Next, the rear-leg knee (joint RT: 76 ms), ankle (joint RT: 109 ms), and hip (joint RT: 111 ms) reacted to the gun signal, generating an extension moment during the stance phase after nearly 0 Nm at the set position. In all joints in both legs, the front-leg knee was the last to react to the gun signal, generating an extension moment during the stance phase (joint RT: 131 ms). Similar extension force-generation patterns within the three joints of the front-leg were observed during the stance phase. The elbow and wrist joint flexion moments were reduced at the moment of take-off, already generating flexion moments at the set position. However, the values were closer to 0 Nm compared to the other joint moments. Whole-body RT for participant #11 was 108 ms in the LL condition. In the other four conditions, similar time-moment curves were observed for participant #11; however, the joint RTs were delayed compared to the LL condition.

As shown by the data for mean ± SEM across all participants, whole-body RT was significantly shorter when the FP length was longer (**Table 1**). In the SS, S, N, L, and LL conditions, whole-body RT were 156 ± 8 ms, 133 ± 6 ms, 125 ± 4 ms, 129 ± 5 ms, and 117 ± 5 ms, respectively. Whole-body RT in the SS condition was significantly longer compared to that in the N, L, and LL conditions; the differences were 31 ± 7 ms, 26 ± 8 ms, and 39 ± 7 ms, respectively.

**Table 1:** Whole-body reaction time (RT) in five foreperiod (FP)-length conditions.

	FP-length condition					F value	P value	Partial η^2^
	SS	S	N	L	LL			
Whole-body RT [ms]	156 ± 8	133 ± 6	125 ± 4^†^	129 ± 5^†^	117 ± 5^†^	33.600	<0.001	0.302

*SS, S, N, L, and LL conditions were conditions whose FP lengths were 1.465, 1.622, 1.780, 1.938, and 2.096 s, respectively. ^†^shows significant difference from SS condition (*P* < 0.05)*.

A two-way repeated-measures ANOVA found that there was a significant FP length × joint interaction on joint RT [*F*(11,228) = 2.234, *P* < 0.05]. In the front-leg hip and ankle; rear-leg hip, knee and ankle; front-arm shoulder and elbow; and rear-arm shoulder, elbow and wrist joints, the joint RT was significantly shorter in the longer FP-length conditions (**Table 2**). In contrast, there were no significant changes in joint RT in the front-leg knee and front-arm wrist among the five FP-length conditions.

**Table 2:** Joint RT in each joint in each upper and lower limb in the five FP-length conditions.

Joint RT [ms]	FP-length condition					*F*-value	*P*-value	Partial η^2^
	SS	S	N	L	LL			
Front-leg								
Hip	168 ± 6	150 ± 4^†^	152 ± 5^†^	142 ± 4^†^	146 ± 8	8.432	<0.01	0.237
Knee	214 ± 13	178 ± 13	162 ± 14	171 ± 2	174 ± 16	2.828	0.109	0.152
Ankle	210 ± 15	186 ± 13	177 ± 16	162 ± 8^†^	163 ± 12^†^	11.993	<0.01	0.241
Rear-leg								
Hip	170 ± 9	157 ± 4	150 ± 6	149 ± 7	145 ± 5	7.294	<0.05	0.160
Knee	165 ± 12	155 ± 5	133 ± 4#	155 ± 5	133 ± 7#	4.565	<0.05	0.152
Ankle	170 ± 9	161 ± 9	149 ± 7^†^	148 ± 10^†^	145 ± 7^†^#	7.020	<0.001	0.270
Front-arm								
Shoulder	164 ± 12	143 ± 8^†^	121 ± 10^†^	118 ± 8^†^#	99 ± 7^†^#	36.881	<0.001	0.348
Elbow	165 ± 14	142 ± 10	142 ± 10	121 ± 8^†^	117 ± 9^†^§	14.745	<0.001	0.235
Wrist	155 ± 14	142 ± 6	134 ± 12	137 ± 6	143 ± 11	0.026	0.348	0.044
Rear-arm								
Shoulder	172 ± 13	145 ± 9^†^	116 ± 8^†^#	111 ± 9^†^#	98 ± 9^†^#	14.266	<0.001	0.429
Elbow	172 ± 12	145 ± 9^†^	125 ± 9^†^#	120 ± 9^†^	113 ± 10^†^	18.890	<0.001	0.350
Wrist	176 ± 16	155 ± 11	150 ± 12	146 ± 9	134 ± 9	5.119	<0.05	0.113

*SS, S, N, L, and LL conditions were conditions whose FP lengths were 1.465, 1.622, 1.780, 1.938, and 2.096 s, respectively. ^†^, #, and §show significant difference from the SS, S, and N conditions, respectively (*P* < 0.05)*.

In the SS condition, positive relationships of whole-body RT were significantly observed between front-leg hip and knee joint RT, rear-leg knee and ankle joint RT, front-arm wrist RT and rear-arm shoulder and wrist joint RT (**Table 3**). In contrast, in the S condition, a positive relationship was observed between only the front-arm shoulder joint RT and, in the N, L, and LL conditions, no significant relationships were observed between whole-body RT and joint RT. Stepwise multiple regression analysis clarified that the rear-arm wrist joint RT was closely to whole-body RT in the SS condition, and the front-arm shoulder joint RT was closely related to whole-body RT in the S condition (**Table 4**). In contrast, in the N, L, and LL conditions, no joint RT was found to be closely related to whole-body RT.

**Table 3:** Relationships between the whole-body RT and joint RT in the five FP-length conditions.

Whole-body RT vs.	FP-length condition				
	SS	S	N	L	LL
Front-leg					
Hip joint RT	0.578^∗∗^	0.224	-0.110	-0.110	-0.025
Knee joint RT	0.484^∗^	0.150	0.386	0.339	-0.021
Ankle joint RT	0.195	-0.008	0.037	-0.134	0.050
Rear-leg					
Hip joint RT	0.436	-0.103	0.013	-0.033	-0.334
Knee joint RT	0.478^∗^	0.200	-0.027	0.292	-0.073
Ankle joint RT	0.578^∗∗^	0.014	-0.036	0.027	-0.036
Front-arm					
Shoulder joint RT	0.279	0.581ˆ**	-0.302	-0.121	0.098
Elbow joint RT	0.392	0.313	0.046	-0.114	-0.118
Wrist joint RT	0.487^∗^	0.296	-0.107	-0.108	-0.096
Rear-arm					
Shoulder joint RT	0.445^∗^	0.326	-0.089	-0.077	-0.125
Elbow joint RT	0.294	0.039	-0.068	-0.105	-0.435
Wrist joint RT	0.620^∗∗^	-0.122	0.179	-0.133	-0.322

*SS, S, N, L, and LL conditions were conditions whose FP lengths were 1.465, 1.622, 1.780, 1.938, and 2.096 s, respectively. ^∗^ (*P* < 0.05) and ^∗∗^ (*P* < 0.01) show significant correlation*.

**Table 4:** Stepwise multiple regression analyses to identify joint RT which was closely related to whole-body RT in the five FP-length conditions.

Closely related joint RT	*B*	SEB	β	*t*	*p*	*R*^2^
SS condition						0.384
Rear-arm wrist joint RT	0.297	0.088	0.620	3.353	< 0.01	
S condition						0.337
Front-arm shoulder joint RT	0.425	0.141	0.581	3.025	< 0.01	
N condition	No joint RT were selected					
L condition	No joint RT were selected					
LL condition	No joint RT were selected					

*SS, S, N, L, and LL conditions were conditions whose FP lengths were 1.465, 1.622, 1.780, 1.938, and 2.096 s, respectively. B, unstandardized regression coefficient; SEB, standard error of the unstandardized regression coefficient; A, standardized regression coefficient*.

## Discussion

In this study, the effect of variable FP length on whole-body RT was first investigated in the dynamic initial motion of experienced sprinters. Our main finding was that the shorter FP in the block start signal, which was based on a 100-m athletic race, led to longer whole-body RT and changed multiple-joint coordinated motion. Therefore, our main hypothesis was supported.

Previous studies conducted block start experiments using 2.0 s or 3.0-4.0 s FP lengths of the start signal (Pain and Hibbs, 2007; Brown et al., 2008). The FP length was 1.780 ± 0.017 s (SD 0.158 s) in recent international athletic games and was less than that reported in previous studies (Pain and Hibbs, 2007; Brown et al., 2008). The FP range for the present experiment was 0.632 s (ranged from 1.465 to 2.096 s). This FP range can be considered as a very short absolute value in a simple reaction task compared to that used in basic research (Elliott, 1973; Niemi, 1979; Boulay et al., 2011). However, the shorter FP length led to longer whole-body RT. This suggests that FP influences the subsequent RT enough if its length and range are short, as in an experiment such as that in our study.

The absolute value of RT in block starts has been reported in many previous studies (e.g., Henry, 1952; Gutiérrez-Dávila et al., 2006; Lipps et al., 2011). Brown et al. (2008) used a 2.0-s FP length and 120-dB sound intensity for the gun signal, which was similar to our LL condition, and reported that the RT was 120 ± 20 ms (mean ± SD). This RT corresponded to the RT of 117 ± 5 ms in the LL condition in this study, whose FP length was 2.096 s, which seems to confirm the validity of our result. In a competitive race, RT was 166 ± 30 ms (mean ± SD) for male sprinters (Tønnessen et al., 2013). This RT was longer than whole-body RT in any of the conditions. However, this RT in a previous study (Tønnessen et al., 2013) was influenced not only by changes in FP length (Niemi, 1979; Niemi and Näätänen, 1981) but also by differences in performance-level categories (Tønnessen et al., 2013), sound intensity of gun signal (Brown et al., 2008) and so on. Particularly, one possible reason for the difference in whole-body RT compared to a previous study (Tønnessen et al., 2013) is that our detection threshold for RT was lower because of different GRF filtering. Filtering information was not provided for the detection threshold used in the previous study (Tønnessen et al., 2013); hence, unfortunately, we cannot accurately compare whole-body RT between the studies.

In block start, a shorter FP length led to a longer whole-body RT. This results did not correspond with the findings of Cressman et al. (2006). Cressman et al. (2006) clarified that an intended motion is programmed before the arrival of the startle stimulus within 2.5 s of the warning stimulus. In contrast, FP lengths in all conditions were shorter than those in the previous study (Cressman et al., 2006). In addition, in block start, extending legs for the crouched ‘set’ position is involved during the FP, and sprinters are forced to conduct an additional motor program during the FP. Therefore, it is assumed that a psychological refractory period occurred in the shorter FP-length condition, which delayed whole-body RT. In fact, in only SS condition, some sprinters who delayed joint RT in approximately half of the 12 joints had delayed whole-body RT. Thus, short FP lengths may affect sprinters by not allowing them to prepare an acceleration motor program in block start.

Whole-body RT emerges from coordinated movement from force generation in multiple joints of all limbs. A two-way repeated-measures ANOVA showed a significant FP length × joint interaction in joint RT, showing that a multi-joint coordination pattern is changed based on the gunfire timing. Longer FP length did not influence front-leg knee and front-wrist joint RT, while it did influence shorter joint RT in the other joints. In a few studies, RT values produced a U-shaped curve with an optimal FP length (Yuanhui and Kasai, 1993; Cuisinier et al., 2007). Similarly, the front-leg knee and front-arm wrist joint RT created a U-shaped curve; longer these joint RT were not sensitively decreased in the L and LL conditions. Unfortunately, we cannot speculate on the specific mechanism explaining why joint RT in the front-leg knee and front-arm wrist was not linearly affected by FP length in this study. However, in the N condition, the front-leg knee (joint RT: 162 ± 14 ms) and front-arm wrist (joint RT: 134 ± 12 ms) did not react earlier to the imperative signal rather than the other joints and were longer than whole-body RT (125 ± 4 ms). Moreover, the stepwise multiple regression analysis in the N condition showed that no joint RT was closely related to whole-body RT. This may indicate that, for many sprinters, front-leg knee and front-arm wrist motions are not the main contributors to whole-body RT under the normal gunfire timing.

Stepwise multiple regression analyses clarified that joint RT that were closely related to whole-body RT changed in accordance with the FP-length condition. In the SS condition, only a rear-arm wrist joint RT was chosen as being closely related to whole-body RT. However, the rear-arm wrist joint RT (176 ± 16 ms) was longer than whole-body RT (156 ± 8 ms) in the SS condition. Similarly, in the S condition, the front-arm shoulder joint RT (143 ± 8 ms), which was a closely related joint RT to whole-body RT, was longer than whole-body RT (133 ± 6 ms). Therefore, even though cross-sectional relationships were observed between whole-body RT and closely related joint RT under shorter gunfire timings, no main contributor for whole-body RT is probably involved for many sprinters. In the N, L, and LL conditions, whole-body RT was significantly related to any joint RT to begin with. These findings suggest that multi-joint coordination of dynamic beginning motion is changed by the unanticipated timing of loud auditory stimuli and the main joint determinant for whole-body RT is based on the individuality of sprinters.

At a set position, relative to the whole-body center of mass, the contacting hands anteriorly and feet posteriorly leads sprinters to maintain stability. The position of shoulders being forwarded at set position generating the flexion moment, arms allows crutch (Slawinski et al., 2010). After the hands are released from the ground with initially reducing the shoulder flexion moments, only the feet actually contact the ground and the whole-body center of mass leans forward relative to the foot contact positions. This forward-leaned position is advantageous for anteriorly accelerating the whole-body quickly (e.g., Kugler and Janshen, 2010; Morin et al., 2011, 2012). Therefore, releasing the hand from ground early can be considered as a key motion in block start. As highlighted above, in the SS and S conditions, only the upper-limb joint RT was related to whole-body RT, and the upper-limb joint RT was longer than whole-body RT. This suggests that shortening whole-body RT contribute to the subsequent quick key motion of arms in block start under shorter gunfire timings.

Shoulder joints, which are located in the most proximal position to the central nerve system, reacted first to the imperative signal more than other joints in the N, L, and LL conditions. Interestingly, in the LL conditions, the shoulder joint initially reacted within 100 ms. This suggests that shoulder joints of both arms more sensitively contribute to whole-body RT rather than the other joints in the longer FP-length conditions. As shown for participant #11, whose front-arm shoulder joint RT was 61 ms, it can be considered that the shoulder joints of some sprinters react very quickly to a long gunfire timing. Thus, there is a possibility that participant #11 may have anticipated the go signal. Fortunately, whole-body RT of participant #11 was slightly over 100 ms; however, this results may indicate that, for some sprinters exposed to a longer gunfire timing, this initial shoulder motion might lead to a false start motion in competitive races.

The difference in whole-body RT lengths between the shortest- and longest-RT length conditions, the SS and LL conditions, was 39 ± 7 ms, corresponding to the RT difference in previous studies with similar FP lengths (Drazin, 1961; Yuanhui and Kasai, 1993). This suggests that, under current regulations, which allow the starter to determine the timing of the gun-signal subjectively, the RT length and the subsequent finish time in a 100-m race must differ race by race. Therefore, for managing athletic games, our results can recommend to treat either of the following two methods for start signals at sprint running events. First, FP length in the race should be recorded in addition to wind speed during the race. This will allow sprint running ability to be distinguished even if the finish times are the same across multiple sprinters in different races. Second, if possible, the FP length in all block starts should be fixed, for instance, at 1.769 s, as in the N condition. This could be achieved by using a computer system that automatically controls gunfire timing. Of course, under the fixed FP-length condition, anticipation of the gunfire timing will occur; therefore, catch trials that do not provide the imperative signal will be needed with an instruction that athletes attempt not to react within 100 ms. Further research is required to investigate how the catch trials should be involved in the computer system and if they are valid in an actual competitive situation. Both would reduce the effect of FP length on whole-body RT in block starts and will likely facilitate fairness in block starts after gunfire across races.

This study has three limitations. First, the auditory data of the ‘set’ and gunfire in the athletic event were captured using that in television broadcasts, and thus we could not identify how the starter’s voice and gunfire were recorded. Therefore, the FP length that we measured likely involve some error compared to that which sprinters actually heard in the athletic event. Second, the number of trials was limited to one trial in each condition. We could not clarify RT variability in each individual sprinter; therefore, the RT of some participants may involve measurement error. However, according to a previous study (Bradshaw et al., 2007), the number of experimental trials should not be large because sprinters cannot perform sprint starts consecutively with maximal effort. Therefore, we had to select the limited number of trials. Third, we did not measure whole-body EMG activity during the block start. Joint moment is produced by muscle activity around the joint. Previous EMG studies showed that the muscle activities that first associate with the onset of the pushing block start are consistent with individual differences among sprinters (Mero and Komi, 1990; Komi et al., 2009). However, muscle activity in the arms and legs could not be measured simultaneously because of the limited number of electrodes. Despite these limitations, our findings may provide us with an understanding of the effect of variable FP length on subsequent whole-body RT in block starts.

## Conclusion

This study demonstrated that long FP length, as determined by FP length in athletic games over the last 5 years, affects the subsequent short joint moment and shorter whole-body RT in block start. This information could lead to new methods for start signals in sprint running events and could explain the fundamental effect of anticipatory RT in whole-body reaction motion in humans.

## Author Contributions

Conceived and designed the experiments: MO and TK. Performed experiments: MO and TK. Analyzed data: MO. Interpreted results of research: MO, TK, and TI. Drafted manuscript and prepared tables/figures: MO and TK. Edited, critically revised paper and approved final version of manuscript: MO, TK, and TI.

## Conflict of Interest Statement

The authors declare that the research was conducted in the absence of any commercial or financial relationships that could be construed as a potential conflict of interest.

## References

[B1] Ae M. Tang H. Yokoi T. (1992). Estimation of inertia properties of the body segments in Japanese athletes. *Biomechanism* 11 23–33. 10.3951/biomechanisms.11.23

[B2] Bell A. L. Pedersen D. R. Brand R. A. (1990). A comparison of the accuracy of several hip center location prediction methods. *J. Biomech.* 23 617–621. 10.1016/0021-9290(90)90054-7 2341423

[B3] Boulay C. B. Sarnacki W. A. Wolpaw J. R. McFarland D. J. (2011). Trained modulation of sensorimotor rhythms can affect reaction time. *Clin. Neurophysiol.* 122 1820–1826. 10.1016/0021-9290(90)90054-7 21411366 PMC3132832

[B4] Bradshaw E. J. Maulder P. S. Keogh J. W. L. (2007). Biological movement variability during the sprint start: performance enhancement or hindrance? *Sports Biomech.* 6 246–260. 10.1080/14763140701489660 17933190

[B5] Brown A. M. Kenwell Z. R. Maraj B. K. V. Collins D. F. (2008). “Go” signal intensity influences the sprint start. *Med. Sci. Sports Sci.* 40 1144–1150. 10.1249/MSS.0b013e31816770e1 18460990

[B6] Cressman E. K. Carlsen A. N. Chua R. Franks I. M. (2006). Temporal uncertainty does not affect response latencies of movements produced during startle reactions. *Exp. Brain Res.* 171 278–282. 10.1007/s00221-006-0459-x 16604311

[B7] Cuisinier R. Olivier I. Nougier V. (2007). The increased foreperiod duration to attain the neutral optimal preparation from sitting to standing. *Exp. Brain Res.* 180 321–331. 10.1007/s00221-007-0862-y 17265039

[B8] Drazin D. (1961). Effects of foreperiod, foreperiod variability, and probability of stimulus occurrence on simple reaction time. *J. Exp. Psychol.* 62 43–50. 10.1037/h0046860 13724295

[B9] Elliott R. (1973). Some confounding factors in the study of preparatory set in reaction time. *Mem. Cogn.* 1 13–18. 10.3758/BF03198063 24214471

[B10] Gutiérrez-Dávila M. Dapena J. Campos J. (2006). The effect of muscular pre-tensing on the sprint start. *J. Appl. Biomech.* 22 194–201. 10.1123/jab.22.3.194 17215551

[B11] Henry F. M. (1952). Force-time characteristics of the sprint start. *Res. Q.* 23 301–308.

[B12] Komi P. V. Ishikawa M. Salmi J. (2009). IAAF sprint start research project: is the 100 ms limit still valid? *New Stud. Athl.* 24 37–47.

[B13] Kugler F. Janshen L. (2010). Body position determines propulsive forces in accelerated running. *J. Biomech.* 43 343–348. 10.1016/j.jbiomech.2009.07.041 19863962

[B14] Lipps D. B. Galecki A. T. Ashton-Miller J. A. (2011). On the implications of a sex difference in the reaction times of sprinters at the Beijing Olympics. *PLoS ONE* 6:e26141 10.1371/journal.pone.0026141 PMC3198384 22039438

[B15] Maslovat D. Carlsen A. N. Ishimoto R. Chua R. Franks I. M. (2008). Response preparation changes following practice of an asymmetrical bimanual movement. *Exp. Brain Res.* 190 239–249. 10.1007/s00221-008-1467-9 18592228

[B16] Mero A. Komi P. V. (1990). Reaction time and electromyographic activity during a sprint start. *Eur. J. Appl. Physiol.* 61 73–80. 10.1007/BF00236697 2289501

[B17] Mero A. Kuitunen S. Harland M. Kyröläinen H. Komi P. V. (2006). Effects of muscle-tendon length on joint moment and power during sprint starts. *J. Sports Sci.* 24 165–173. 10.1080/02640410500131753 16368626

[B18] Morin J. B. Bourdin M. Edouard P. Peyrot N. Samozino P. Lacour J. R. (2012). Mechanical determinants of 100-m sprint running performance. *Eur. J. Appl. Physiol.* 112 3921–3930. 10.1007/s00421-012-2379-8 22422028

[B19] Morin J. B. Edouard P. Samozino P. (2011). Technical ability of force application as a determinant factor of sprint performance. *Med. Sci. Sports Exerc.* 43 1680–1688. 10.1249/MSS.0b013e318216ea37 21364480

[B20] Niemi P. (1979). Stimulus intensity effects on auditory and visual reaction processes. *Acta Psychol.* 43 299–312. 10.1016/0001-6918(79)90038-6 495172

[B21] Niemi P. Näätänen R. (1981). Foreperiod and simple reaction time. *Psychol. Bull.* 89 133–162. 10.1037/0033-2909.89.1.133

[B22] Otsuka M. Kurihara T. Isaka T. (2015). Effect of a wide stance on block start performance in sprint running. *PLoS ONE* 10:e0142230 10.1371/journal.pone.0142230 PMC4636312 26544719

[B23] Otsuka M. Shim J. K. Kurihara T. Yoshioka S. Nokata M. Isaka T. (2014). Effect of expertise on 3D force application during the starting block phase and subsequent steps in sprint running. *J. Appl. Biomech.* 30 390–400. 10.1123/jab.2013-0017 24615252

[B24] Pain M. T. G. Hibbs A. (2007). Sprint starts and the minimum auditory reaction time. *J. Sports Sci.* 25 79–86. 10.1080/02640410600718004 17127583

[B25] Slawinski J. Bonnefoy A. Levêque J. M. Ontanon G. Riquet A. Dumas R. (2010). Kinematic and kinetic comparisons of elite and well-trained sprinters during sprint start. *J. Strength Cond. Res.* 24 896–905. 10.1519/JSC.0b013e3181ad3448 19935105

[B26] Telford C. W. (1931). The refractory phase of voluntary and associative response. *J. Exp. Psychol.* 14 1–36. 10.1037/h0073262

[B27] Tønnessen E. Haugen T. Shalfawi A. I. (2013). Reaction time aspects of elite sprinters in athletic world championships. *J. Strength Cond. Res.* 27 885–892. 10.1519/JSC.0b013e31826520c3 22739331

[B28] Winter D. A. (2009). *Biomechanics, and Motor Control of Human Movement*. Hoboken, NJ: Wiley, and Sons 10.1002/9780470549148

[B29] Yuanhui M. Kasai T. (1993). Effects of foreperiod on response latency of different movement patterns. *Percept. Mot. Skill* 77 1160–1162. 10.2466/pms.1993.77.3f.1160 8170765

